# Activated HGF-c-Met Axis in Head and Neck Cancer

**DOI:** 10.3390/cancers9120169

**Published:** 2017-12-12

**Authors:** Levi Arnold, Jonathan Enders, Sufi Mary Thomas

**Affiliations:** 1 Departments of Otolaryngology, University of Kansas Medical Center, Kansas City, KS 66160, USA; larnold6@kumc.edu (L.A.); j255e970@kumc.edu (J.E.); 2 Anatomy & Cell Biology, University of Kansas Medical Center, Kansas City, KS 66160, USA; 3 Cancer Biology, University of Kansas Medical Center, Kansas City, KS 66160, USA

**Keywords:** receptor tyrosine kinase, c-Met, hepatocyte growth factor, tumor microenvironment, head and neck squamous cell carcinoma

## Abstract

Head and neck squamous cell carcinoma (HNSCC) is a highly morbid disease. Recent developments including Food and Drug Administration (FDA) approved molecular targeted agent’s pembrolizumab and cetuximab show promise but did not improve the five-year survival which is currently less than 40%. The hepatocyte growth factor receptor; also known as mesenchymal–epithelial transition factor (c-Met) and its ligand hepatocyte growth factor (HGF) are overexpressed in head and neck squamous cell carcinoma (HNSCC); and regulates tumor progression and response to therapy. The c-Met pathway has been shown to regulate many cellular processes such as cell proliferation, invasion, and angiogenesis. The c-Met pathway is involved in cross-talk, activation, and perpetuation of other signaling pathways, curbing the cogency of a blockade molecule on a single pathway. The receptor and its ligand act on several downstream effectors including phospholipase C gamma (PLCγ), cellular Src kinase (c-Src), phosphotidylinsitol-3-OH kinase (PI3K) alpha serine/threonine-protein kinase (Akt), mitogen activate protein kinase (MAPK), and wingless-related integration site (Wnt) pathways. They are also known to cross-talk with other receptors; namely epidermal growth factor receptor (EGFR) and vascular endothelial growth factor receptor (VEGFR) and specifically contribute to treatment resistance. Clinical trials targeting the c-Met axis in HNSCC have been undertaken because of significant preclinical work demonstrating a relationship between HGF/c-Met signaling and cancer cell survival. Here we focus on HGF/c-Met impact on cellular signaling in HNSCC to potentiate tumor growth and disrupt therapeutic efficacy. Herein we summarize the current understanding of HGF/c-Met signaling and its effects on HNSCC. The intertwining of c-Met signaling with other signaling pathways provides opportunities for more robust and specific therapies, leading to better clinical outcomes.

## 1. Introduction

Of all the neoplasms in the head and neck region, roughly 90% are classified as head and neck squamous cell carcinoma (HNSCC) [1]. The estimated new cases for HNSCC related deaths in the United States alone is nearly 50,000 [2]. Even with major advances in multimodality treatment, the five-year progression-free survival rate in HPV negative patients is less than 50% and overall survival rate for recurrent or metastatic HNSCC patients remains necessitous [3]. Major risk factors associated with HNSCC occurrence include human papilloma virus (HPV) infection, excessive alcohol consumption, tobacco use, chewing areca nut, poor oral hygiene, and genetic alterations leading to susceptibility to malignancies [4,5]. Treatment is stage and site dependent. Irradiation, chemotherapy, and surgery are the current therapeutic options.

Cetuximab, a human/mouse chimeric monoclonal antibody targeting (EGFR), is the only FDA-approved targeted therapy for EGFR in HNSCC [6]. However, combinatorial cetuximab treatment with radiation or chemotherapy yields only modest results (29.3 vs. 49 months and 7.4 vs. 10.1 months, respectively) [7]. Despite a growing body of evidence that suggest HNSCC is highly heterogeneous [8], EGFR overexpression has been identified as common in up to 90% of HNSCCs [9]. Resistance to cetuximab has been observed relative to hepatocyte growth factor/mesenchymal-epithelial transition factor (HGF/c-Met) signaling [10]. Immune-checkpoint inhibitors (that facilitate T-cell activation), nivolumab and pembrolizumab promise greater overall survival and extended time between recurrence [11]. Both agents were Food and Drug Administration (FDA) approved for advanced HNSCC in 2016. Although immune-checkpoint inhibitors point to the promise of targeted therapies in HNSCC, early data from clinical trials indicate limited efficacy with monotherapy in HNSCC compared to standard chemotherapy. Thus, there continues to be a need for more effective approaches for HNSCC. Receptor tyrosine kinases regulate pro-tumor signaling pathways in HNSCC and are an attractive therapeutic target.

The c-Met proto-oncogene is a membrane spanning receptor tyrosine kinase (RTK) whose ligand is the hepatocyte growth factor (HGF) [12]. Its aberrant activity is documented in HNSCC as well as many other cancers [13]. Its maladaptive signaling in HNSCC is associated with invasion, metastasis, and intertwining with other pathways. Met expression is associated with worse prognosis and lower overall survival [14]. Further, HGF/Met expression levels have been clinically shown to be inversely proportional to patient survival with late-stage nasopharyngeal carcinoma [15]. This review summarizes the current knowledge about HGF/c-Met pathway, as it is associated with HNSCC, and posits that c-Met is a valuable target in the treatment therein.

## 2. Brief HGF/c-Met History

Hepatocyte growth factor, also then named “Hepatotropin”, was isolated from serum in partially hepatectomized rats and described as a mitogen of liver regeneration in 1984 [16]. HGF was purified from rat platelets in 1984 [17] human plasma in 1988 [18,19] and rat liver in 1991 [20].

Scatter factor (SF) was first identified as a protein secreted by fibroblasts and smooth muscle and had properties that dispersed “scattered” uniform epithelial cells in culture by disrupting intracellular junctions [21,22,23]. Until the early 1990s, HGF and SF once thought to be two different ligands until they were discovered to be, in fact, identical structures serving the same receptor, c-Met [12,24,25,26,27,28,29]. Around the same time, HGF was being implicated as a mitogen, motogen, and morphogen (having the ability to promote mitosis, motility, morphological changes, respectively) [30], while also being recognized as an elicitor of angiogenesis [31], tumorigenesis [32], and invasion [23]. Independently, NIH 3T3 fibroblasts transfected with human *Met* cDNA displayed motogenic and invasive properties when treated with HGF [33].

HGF displayed cytotoxic effects in a few cell lines, deviating from known HGF activity [34]. With this in mind, Higashio et al. found that Sarcoma-180 was killed in the presence of soluble factors derived from human lung fibroblasts naming it tumor cytotoxic factor (TCF). The same group found the molecular makeup of TCF and HGF to be identical [35,36,37,38].

c-Met, the receptor for HGF, was originally discovered in osteosarcoma cell line that was chemically transformed in 1984 [39]. The chemical treatment yielded an oncogenic activation wherein chromosome 1 was rearranged and fused a region called *tpr* (translocated promoter region) [40,41,42]. Tpr-Met is highly constitutively active as a tyrosine kinase and can alter cells in vitro. Tpr-Met cDNA led to the identification of the full length c-Met receptor [43].

c-Met was shown to be overexpressed in the human gastric cancer line (GTL-16), and later in human thyroid carcinomas, human colorectal cancer, and with many other epithelial tumors in the early 90s [44,45,46]. Pertinent to this review, Matsumoto et al. showed that soluble factors from fibroblast conditioned media promoted invasion of HNSCC [47]. The group later identified the factors as HGF/SF in 1994 [48]. In 2000, the first report of direct MET involvement of tumor metastasis (found in HNSCC, but it was the first time the connection between MET and tumor growth was made) was noted by Di Renzo et al. in. They saw that clonal expansion of neoplastic cells harbored mutations that activated MET [49].

## 3. Role of HGF/c-Met Signaling in Homeostasis

In normal development and tissue regeneration HGF/c-Met is crucial. HGF/c-Met has physiological roles in cardiovascular remodeling [50], skeletal muscle regeneration [51], differentiation and migration during spermatogenesis [52], axonal growth [53], renal tubulogenesis [54], and blood cell growth and differentiation [55]. Recently, HGF has been found to be elevated during cerebral aneurism promoting healing of tissue damage, and promotes organ regeneration. Blood drawn from 16 patients suffering intracranial aneurysms was drawn from either aneurysm lumen or femoral artery. There was a five-fold increase in HGF plasma levels drawn from the site of injury. The same study evaluated mice with induced intracranial aneurism treated with a c-Met antagonist or control. The c-Met antagonist treated mice experienced decreased survival compared to control [56].

Loreto et al. (2010) studied c-Met expression during salivary gland morphogenesis. A sample of 12 human embryonic salivary glands were studied at different stages of development using immunohistochemical staining. While all stages of salivary gland development demonstrated staining for c-Met, there was stronger expression in the earlier stages of development [57]. HGF/c-Met has been studied in the embryogenesis of liver development [58], tooth development [59], mammary gland morphogenesis [60,61,62], skeletal myogenesis [14], neural induction [63], chemo attraction of motor neurons [64,65,66], and hepatic bile duct formation [67]. It is also involved in regulation of differentiating events during development [68,69,70].

The study of cancer compared to wound healing and tissue regeneration has long been thought as apposite, with many parallels being readily identified. Additionally, many cancer therapies interrupt wound healing [71]. Likewise, many signals in normal embryogenesis are dysregulated in cancers [72]. HGF/c-Met is important to both normative function and cancer biology, suggesting a value in appreciating both.

## 4. Ligand Expression, Secretion, and Activation

**Figure 1:**
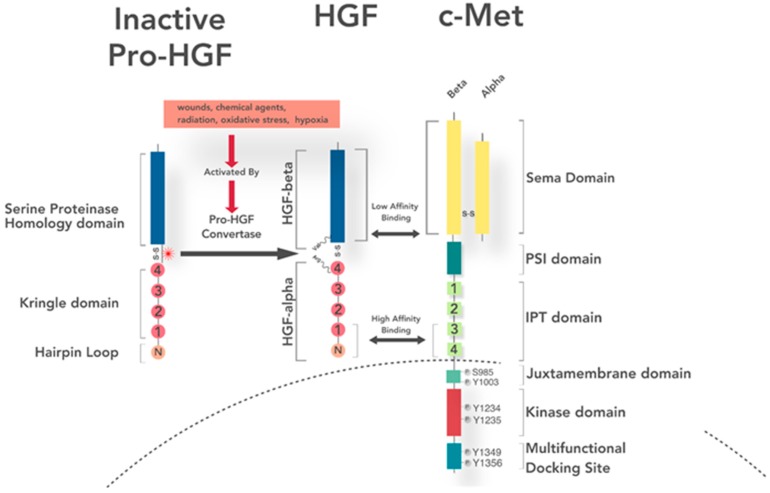
Pro-HGF (hepatocyte growth factor) is secreted as an inactive, single-chain precursor. Activation is stimulated by wounds, chemical agents, radiation, hypoxia, and oxidative stress among other cellular disruptions. It is activated by cleavage of the bond at Arg494-Val495 by a cellular protease to yield active, heterodimeric HGF with α (consisting of four Kringle domains and a hairpin loop) and β-chains linked by a disulfide bond. Extracellularly, the c-Met receptor consist of the sema domain (with disulfide linked α-β domains), the PSI (Plexin, Semaphorin, and Integrin) domain and four IPT (Ig-like, plexins, transcription factors) domains. Intracellular mesenchymal–epithelial transition factor (c-Met) domains include a kinase domain flanked by the juxtamembrane domain and the multifunctional docking site. High affinity binding occurs between the hairpin loop and first Kringle domain of the α-chain of HGF and the third and fourth IPT domain of c-Met. Low affinity binding occurs between the β-chain of HGF and the sema domain of c-Met.

HGF expression is normally found in the vicinity of epithelial and endothelial cells expressing the c-Met receptor. While much remains to be discovered about HGF formation and secretion, it has been reported that granulocytes (particularly neutrophils) store inactive pro-HGF [73], and coagulation factor Xa was shown to induce secretion of mature HGF [74]. HGF is secreted by stromal cells as an inactive 92 kDa single-chain polypeptide known as pro-HGF. Pro-HGF consists of an amino-terminal heparin binding domain, four Kringle domains, and a serine protease-like domain [75]. While pro-HGF can bind c-Met, it does so with no activation of the receptor. Maturation of pro-HGF occurs in the ECM [29]. Pro-HGF is cleaved between the fourth Kringle domain Arg494 and serine proteinase domain Val495 to its active heterodimer by serine proteases [76]. Though pro-HGF can bind to c-Met with low affinity, it does not induce receptor activation. HGF is processed by serum or cellular proteases including HGFA, factor XIIa and XIa, matriptase, hepsin, transmembrane protease serine 13 (TMPRSS13), human airway trypsin-like protease (HAT), urokinase-type plasminogen activator (uPA), and tissue-type plasminogen activator (tPA) [77]. SPINT1 and SPINT2, are genes encoding serine protease inhibitors that inhibit pro-HGF cleavage adding another degree of regulation to the HGF/c-Met signaling axis [78,79]. Additionally, heparan-sulfate proteoglycans (HSPGs) bound to HGF, facilitate the binding of pro-HGF to the extracellular matrix proteins, protect against proteolytic cleavage, create stores for later use, enable clearance by endocytosis, and regulate tissue diffusion [80,81,82]. Normal breast ductal branching depends on HSPGs binding HGF in vivo [83]. In physionormative biology, HSPGs binding HGF seems to protect from aberrant signaling by sequestering and degrading the growth factor [84]. In fact, blocking heparanase, an enzyme that cleaves the HSPG side chain thereby interrupting their affinity for growth factors, actually reduces tumor growth and extends time to recurrence [85,86]. Contrarily, over-sulfated heparan (clinically given as a coagulating agent) can amplify many signaling pathways [87]. Activated HGF consists of an α-chain with an N-terminal, hairpin loop domain linked via disulfide bond to the fourth Kringle domain in the β-chain. The N terminal and first Kringle domain of HGF bind with high affinity to the Ig-like fold, plexins, transcription factor (IPT) domain, suggesting N and K1 as receptor agonist [88]. The SPH domain of HGF binds the sema domain of c-Met with lower affinity, but with the ability to pull two HGF bound c-Met complexes together for dimerization [89] (Figure 1).

## 5. Receptor Structure and Activation

Mesenchymal epithelial transition factor (c-Met) is a semaphorin, membrane spanning receptor tyrosine kinase that is encoded by the MET proto-oncogene [12,90]. From the *met* transcript, a 150 kDa polypeptide is produced that is partially glycosylated to become a 170 kDa precursor protein [91,92]. The Met precursor is additionally glycosylated and cleaved into a 50 kDa α-chain and a 140 kDa β-chain (Figure 1). The larger β-subunit contains an intracellular tyrosine kinase domain, a membrane-spanning domain, and a larger extracellular region. The α-subunit is exclusively extracellular. The two subunits are joined by di-sulfide bonds and together make up a mature α-β Met heterodimer [90,93,94].

The α-chain of mature, bivalent HGF binds c-Met β-chain with high affinity at the immunoglobulin-plexin-transcription (IPT) domain. Meanwhile the β-chain of HGF binds c-Met’s Sema domain with low affinity. Engineered proteins mimicking the IPT domains of Met show anti-tumor effects in mice; in vitro display inhibit HGF induced cell growth and invasion [95]. It is worth noting that pro-HGF binds c-Met with high affinity and can displace active HGF. HGF is diffused in low levels throughout the ECM, while pro-HGF is in higher quantities. HGF is supplied by stromal cells in a paracrine manner and is not controlled at the transcriptional level. High affinity pro-HGF allows its receptor to be “primed” to meet and environmental challenges. Activation can occur while pro-HGF is receptor-bound.

In physionormative settings, binding of HGF activates c-Met by phosphorylating two intracellular tyrosine residues, inducing catalytic activity [96]. The carboxy-terminal of the beta chain has two tyrosine residues that serve as docking sites for intracellular proteins. When phosphorylated, activation of c-Met in response to HGF is partly mediated by autophosphorylation of the two catalytic residues, Tyr1234 and Tyr1235, located within the activation loop of the tyrosine kinase activity receptor [28]. This is followed by phosphorylation of the residues Tyr1349 and Tyr1356 in the C-terminus, activating a docking site for Src homology-2 (SH2) domain, phosphotyrosine binding (PTB) domain, and Met binding domain (MDB) that all contain signal transducers [97,98,99].

### 5.1. Intracellular Signaling

**Figure 2:**
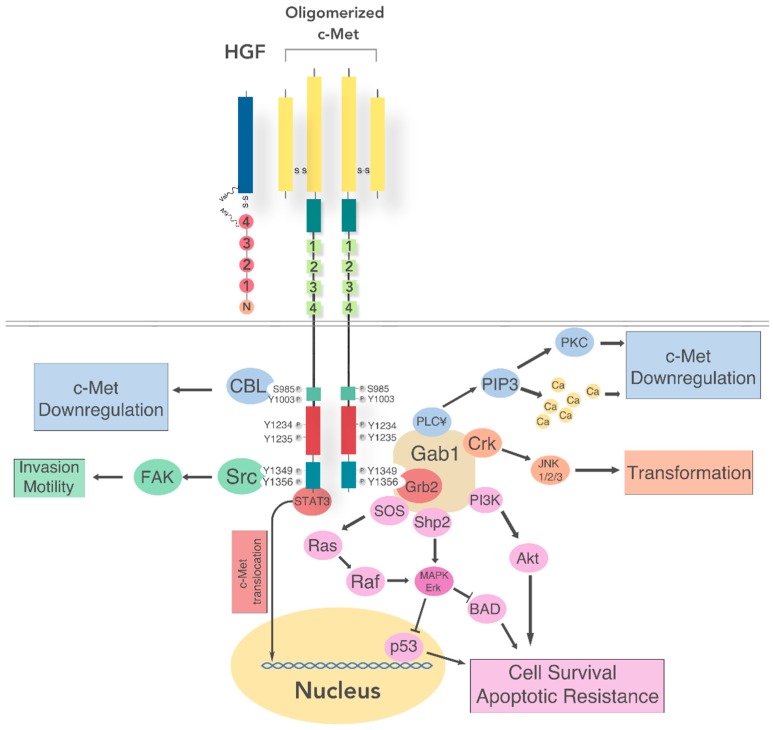
Hepatocyte growth factor (HGF) binding c-Met activates signaling cascades including Src, CT10 (chicken tumor virus No. 10) regulator of kinase (Crk), mitogen-activated protein kinase/ extracellular signal-regulated kinases (Mapk/Erk), and phosphotidylinsitol-3-OH kinase (PI3K), leading to loss of tumor cell apoptosis, survival, transformation, invasion, and motility. Downregulation of c-Met are highlighted in blue. c-Met signaling that promotes cell survival and apoptotic resistance are highlighted in lavender. c-Met signaling that promotes transformation is highlighted in orange. c-Met signaling that promotes invasion and motility is highlighted in green. CBL: casitas B-lineage lymphoma; FAK: focal adhesion kinase; STAT3: signal transducer and activator of transcription-3; PKC: protein kinase c; PIP3:phosphatidylinositol (3,4,5)-trisphosphate; PLC: phospholipase C; JNK: jun n-terminal kinase; Gab1: Grb2-associated adaptor protein; Grb2: growth factor receptor-bound protein 2; Akt: alpha serine/threonine-protein kinase; SOS: Ras guanine nucleotide exchange factor son-of-sevenless; Shp2: Src kinase, and Src homology region-2 containing protein tyrosine phosphatase 2; Ras: rat sarcoma; Raf: rapidly accelerated fibrosarcoma; BAD: Bcl-2 associated death promoter; p53: cellular tumor antigen p53.

Adapter proteins known to interact with the tyrosine residues include: growth factor receptor-bound protein 2 (Grb2), src homology/collagen (SHC), Grb2-associated adaptor protein (Gab1), and CT10 (chicken tumor virus No. 10) regulator of kinase/Crk-like (Crk/CRKL) with signal transducers phosphotidylinsitol-3-OH kinase (PI3K), signal transducer and activator of transcription-3 (Stat3), phospholipase C-γ (PLCγ), Ras guanine nucleotide exchange factor son-of-sevenless (SOS), Src kinase, and Src homology region-2 containing protein tyrosine phosphatase 2 (SHP2) [97,98,99,100,101,102,103,104,105]. Y1349 and Y1356 facilitate interplay with GRB2-associated-binding protein 1 (Gab1), Src, and SHC. Y1356 residue recruits Grb2, PI3K, PLC-γ, and SHP2 to the Met signaling complex [106]. Gab1 is a scaffolding protein adaptor that contains the Met-binding site. The interaction at that site with Met results in the phosphorylation of Gab1, which is responsible for the unique biological effects of HGF [99]. PI3K/AKT (alpha serine/threonine-protein kinase), mitogen-activated protein kinase (MAPK), and signal transducer and activators of transcription (STAT) pathways are downstream pathways affected by normal c-Met signaling (Figure 2).

Activation of MAPK will successively activate different protein kinases whose terminal effectors include extracellular signal-regulated kinases (Erk1 and Erk2), Jun amino-terminal kinases (JNK1, JNK2, and JNK3) and p38 (a class of Mapk’s). These proteins will activate cell cycle regulators producing cell proliferation and stimulating functional alterations in the cytoskeleton, important to cell migration and invasion. Inactivation of B-cell lymphoma 2 (Bcl-2) antagonist of cell death (BAD) and degradation of the pro-apoptotic protein p53 increases cell survival and apoptotic resistance by activation of the PI3K/AKT pathway [107]. STAT3 activation relocates the c-Met receptor to the nucleus from the plasma membrane, and begins activity as a transcription factor regulating genes for cell differentiation and proliferation [108].

The plasma membrane proves to be an important area of complex c-Met interaction with other surface proteins, contributing to dynamic c-Met biological responses—EGFR, α6β4 integrin, semaphorins in the plexin B family, and the variant of the hyaluronan receptor cluster of differentiation 44 (CD44) (links intracellular cytoskeleton and the extracellular matrix) [109,110].

### 5.2. Regulation of c-Met Signaling

After HGF binding, c-Met is endocytosed via clatherin coated vesicles to early peripheral endosomes. This is facilitated by protein kinase Cε (PKCε), that promotes both the transfer of active Erk to focal adhesions and HGF-induced cell migration. Next, PKCα mediates c-Met travel along the microtubule network towards the late perinuclear compartments. The accumulation of c-Met in endosomal compartments near the nucleus is a determining step of STAT3 activation [111].

Downregulation of c-Met involves trafficking and degradation in the lysosomes. Initiated by juxtamembrane domain site Y1003 association with casitas B-lineage lymphoma (CBL) and endocytic adaptors, c-Met then accumulates in multivesicular bodies that subsequently fuse with lysosomes, which then break down the proteins. Loss of Y1003 residue allows c-Met to evade lysomal degradation and become oncogenic [112]. Alternatively, c-Met can also be cleaved in either the extracellular domain or the intracellular domain. Extracellular cleavage occurs by disintegrin and metalloprotease (ADAM) which creates a fragment that sequesters the ligand and obstructs the receptor’s activity. Intracellular cleavage is mediated by γ-secretase that produces a fragment that is destroyed by the proteasome [113].

c-Met downregulation can also occur by its binding to protein tyrosine phosphatases (PTPs), which include receptor-type PTPs density enhanced phosphatase 1 (dEP1) and leukocyte common antigen-related molecule (LAR), and the nonreceptor PTPs PTP1B and T-cell protein tyrosine phosphatase (TCPTP). TCPTP modulates c-Met signaling by dephosphorylating the tyrosines in the kinase domain, and dEP1 downregulates c-Met signaling by dephosphorylating the docking tyrosines [114,115].

## 6. Aberrant Functions in HNSCC

### 6.1. c-MET Drives Tumorigenesis in HNSCC

Typical of most tyrosine kinase receptors, c-MET can be activated by one of three mechanisms. First, ligand binding triggers dimerization and transactivation of the receptor. Phosphorylated c-Met (p-Met) is overexpressed in HNSCC patients. Total c-Met is overexpressed in 78% of HNSCC cases, and 66% of HNSCC demonstrate phosphorylation at activating sites Y1230, Y1234, and Y1235. Activating sites in this case refers to tyrosines whose phosphorylation results in the recruitment of signaling molecules that go on to transduce a signal. Thus the phosphorylation of tyrosine results in an activation of a signaling cascade [116] (Figure 2). Both autocrine and paracrine mechanisms can trigger c-Met signaling. Glioblastoma, osteosarcoma, rhabdomyosarcoma, and breast carcinoma [107,117,118,119] cells demonstrate autocrine activation of the receptor. While HNSCC cell lines express c-Met, they do not secrete HGF. Rather HNSCC cancer-associated fibroblasts (CAF) have been shown to secrete HGF, suggesting an HGF-c-Met paracrine driven tumorigenesis [120].

**Figure 3:**
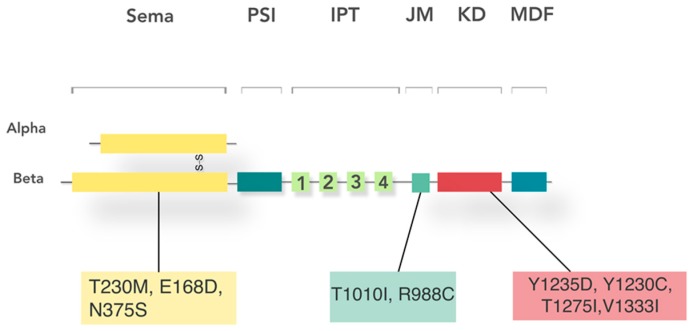
Common c-Met mutations in head and neck squamous cell carcinoma: T230M, E168D, and N375S affect the extracellular sema domain. Intracellularly, T1010I and R988C affect the juxtamedullary (JM) domain, while Y1235D, Y1230C, T1275I, and V1333I affect the kinase domain. PSI: plexin-semaphorin-integrin domain; IPT: immunoglobulin-plexin-transcription domain; KD: kinase domain; MDF: multifunctional docking site.

Second, physical modifications in the receptor can sustain constitutive activation via somatic mutations. In particular, mutations in the kinase domain involving Y1235D and Y1230C are important activators of the c-Met pathway in HNSCC [49,121] (Figure 2). Other germ-line MET mutations occur in liver metastasis from colon cancer [122] and hepatocellular carcinoma [123] cause the gene to be over amplified. MET gene mutations occur in papillary renal cell carcinoma [124], childhood hepatocellular carcinoma [125], and lymph-node metastases of HNSCC [49]. Gain in *MET* copy number is present in 16% of HNSCC cases and is associated with c-Met overexpression and poorer outcomes for those individuals [14]. However, while not at high frequency, *MET* mutations have been identified on several domains including the sema, juxtamembrane, and kinase domains. HNSCC metastatic potential increases with Y1230C and Y1235D kinase domain mutations [49,121]. Y1235D mutation is also associated with impaired local HNSCC tumor control, interferes with response to radiation treatment, and worsens recurrence [126]. In HNSCC tumor tissue ligand binding sema domain sites T230M, E168D, N375S; the juxtamembrane domain sites T1010I and R988C; and the kinase domain sites T1275I and V1333I mutations have been described, but consequences of these mutations not yet determined. While not directly responsible for phorphsorylation, the mutation V1333I was present in 13.5% of HNSCC tumor tissue [116]. Juxtamembrane domain deletion, CBL E3 ubiquitin-ligase recruitment, and exon 14 skipping have been attributed to more rare mutations [127] (Figure 3).

Third, MET protein overexpression is common to thyroid [45,128], ovarian [129,130], pancreatic [131], prostate [132], renal-cell [133], hepatocellular [134], gastric [135], esophageal [136], breast [137], colorectal [138], and head and neck carcinomas [139]. Oligomerization occurs when a cognate, extracellular ligand binds to a monomeric c-Met receptor, stimulating autophosphorylation of the tyrosine residues of the kinase domain. The phosphorylated domain then initiates the c-Met signaling cascade [140]. Ligand overexpression was first reported nearly 20 years ago [141] and has since been shown to drive tumorigenesis in a paracrine manner [120]. HGF overexpression is reported in 58% of HNSCC tumors, lagging behind c-Met expression [10]. This lends to the idea that not only are ligand and receptor overexpression driving tumorigenesis, but also proteins required for concurrent activation are also importantly overexpressed.

### 6.2. c-MET Drives Metastasis in HNSCC

HGF/c-Met plays many roles in the early metastasis of epithelial cancers. These include delamination of epithelial cells and epithelial–mesenchymal transition necessary for non-local epithelial cell migration [142], basement membrane degradation and remodeling by urokinase and matrix metalloproteinase [143], activation of focal adhesion kinase and paxillin causing integrin-dependent migration [48], pre-metastatic niche formation via tumor-derived exosomes [14], tumor lymphangiogenesis [144,145], and hemangiogenesis [146,147].

Lymph node invasion is a common feature of clinical HNSCC and is highly predictive of patient mortality [148]. c-Met is highly expressed in lymph node metastasis in HNSCC, while *MET* gene amplification is low [149]. c-Met tends to be present in all stages of metastasis, it tends to be most expressed at N2 and N3 nodal metastasis [150,151]. HGF is elevated in HNSCC patients compared to healthy individuals [49]. Invasion occurs in normative physiology during embryogenesis. Many of the same regulatory and inductive properties are involved in invasion of cancer. Normal invasive growth signals can be dysregulated contributing to cell transformation and later tumor progression [23]. Mice orthotopically injected with Met knockdown (MetKD) HNSCC cells show no regional lymph node metastasis. Further, these mice show significant reduction in primary tumors and increased cellular apoptosis derived from MetKD [152].

In late stage cancer, primary tumor cells invade contiguous tissues, and some tumor cells travel to distant organs or lymph nodes where they may develop into new, secondary tumors [153]. Anoikis is a process of programmed cell death induced by the detachment of anchorage-dependent cells from their extracellular matrix (ECM) associations. Anchorage independent cell growth and attachment to an inappropriate matrix is typical of metastasis and invasion. Anoikis is the process by which programmed cell death is induced upon detachment from is appropriate matrix (or niche) HGF induces anoikis resistance by fibronectin signaling in HNSCC, disrupting integrin signaling [154]. MET activation prevents anoikis via ERK or Akt pathways. [155] HGF/c-Met signaling may help an abnormal cell survive temporary displacement of focal contacts by PI3K activation, a pro-survival signal [156]. Anoikis resistance is an important mode of action by which HNSCC develops nodal metastasis from the primary tumor site.

## 7. c-MET Pathway Crosstalk in HNSCC

### 7.1. Wnt/β-Catenin

Wnt/β-Catenin is a pathway important for pattern formation during embryogenesis and roles in cancer [157]. β-catenin-dependent transcription is mediated through c-Met in colon cancer cells, and will convert cells to a cell type with cancer stem cell properties [158]. Cancer stem-like (CSC) cells in HNSCC were inhibited with PF-2341066 (a c-Met inhibitor) and β-catenin was shown to be the downregulating factor contributing to CSC elimination. The Wnt pathway frizzled class receptor 8 (FZD8) expression rescued impaired HNSCC cells that were treated with a c-Met inhibitor. It was noted by the same group that FZD8 was upregulated by c-Met signaling through ERK/c-Fos cascade [159].

### 7.2. c-SRC

c-Src regulates many signaling cascades that control various biological outcomes. Inhibition of c-Src in cancer cells can result in anchorage-independent growth, survival, tumor vascularity, migration, metastasis, survival, and invasion. Activated c-Src mediates erlotinib resistance in HNSCC by stimulating c-Met independent of ligand [160]. Sen et al. earlier showed in both xenograft and in vitro models that combination inhibition of c-Met and c-Src resulted in synergistic cytotoxicity, enhanced apoptosis, and decreased tumor size [161].

### 7.3. TGF-β

Bhowmick et al. showed that transforming growth factor (TGF)-β type II receptor knockout in mice gave rise to prostate and gut epithelial tumors by activating c-Met through paracrine overexpression of HGF by stromal cells [162]. TGF-β is mediated by the transcription factor mothers against decapentaplegic homology (SMAD) that bind SMAD binding element (SBE) of target genes, regulating their expression. Smad deletion caused HGF upregulation, contributing to angiogenesis in mice. The HGF promoter of keratinocytes have one such SBE that allows binding of SMAD 1, 2, and 4 families [163]. In HNSCC, in vitro knockdown of Smad4 induced cetuximab resistance by activating TGF-β and c-Met pathways [164].

### 7.4. EGFR

EGFR and c-Met have in common similar downstream pathways: MAPK (RAS/Rapidly accelerating fibrosarcoma (RAF)/ MAP kinase kinases (MEK)/ERK) and PI3K/AKT/mechanistic target of rapamycin (mTOR) [165,166,167,168]. To eliminate these aberrant pathway modalities altogether, dual EGFR/c-Met blockade is of interest. Seiwert et al. showed that dual blockade of SU11274 (c-Met inhibitor) and erlotinib (EGFR inhibitor) in HNSCC lines produce greater-than-additive inhibition of cell growth via erbB3/AKT signaling [116]. Further, Lieu et al. demonstrated that cell lines treated with foretinib and erlotinib or lapatinib (EGFR/human epidermal growth factor (HER) 2 inhibitor) synergistically inhibited growth [169]. Even more, crizotinib (c-Met inhibitor) and gefitinib given in combination potentiated the effects of cell line invasion, wound healing, and proliferation; increased antitumor activity in vivo when compared to EGFR inhibition alone was shown by Xe et al. They also noted that in the absence of HGF, TGF-α (an EGFR ligand) activates c-Met when EGFR is blocked [170]. The intertwining of the RTK’s c-Met and EGFR may explain acquired or constitutive EGFR resistance. c-Met/EGFR co-expression is common. It is likely that c-Met/HGF expression is a common mechanism of EGFR treatment resistance in HNSCC [171]. Wheeler et al. further show that cell lines resistance to cetuximab highly express c-Met, EGFR, HER-2, and HER-3 [172].

## 8. c-MET Contribution to Therapeutic Resistance in HNSCC

The c-Met signaling axis has been implicated in acquired resistance to epidermal growth factor receptor (EGFR) targeting therapies in HNSCC. EGFR, like c-Met, is an RTK that is overexpressed in roughly 90% of HNSCC patients. EGFR overexpression correlates with regional lymph node metastasis and poor outcomes [173]. Acquired EGFR resistance and recurrence secondary to c-Met activation is a common feature in clinical trials [160,174]. Small molecule tyrosine kinase inhibitors (TKI’s), gefitinib or erlotinib, competitively bind the ATP-binding region EGFR, thereby selectively inhibiting the receptor function. Despite EGFR overexpression and the small molecule TKI’s in vitro effectiveness, it does not significantly improve clinical outcomes [175]. c-Met upregulation is also linked to cetuximab resistance in HNSCC patients [10]. EGFR only rarely exhibits activating mutations. Implicatively, EGFR resistance comes not from EGFR itself, but rather from co-activation of other RTKs [176]. c-Met knockdown in vitro sensitizes EGFR to erlotinib, and c-Met inhibition also shows erlotinib sensitization in a dominant-active c-Src subset of EGFR overexpressing HNSCC lines [160].

## 9. Co-Therapies in HNSCC Targeting c-Met

c-Met plays a major role in compensating for inhibition of RTK pathways that drive proliferation and metastasis in HNSCC. Therefore, targeting c-Met in conjunction with these pathways may lead to more effective therapeutic strategies. Here we discuss c-Met inhibitors used in combination with other targeted and nontargeted therapies in clinical and preclinical models of HNSCC.

### 9.1. Crizotinib (PF2341066)

Crizotinib is a class Ia small molecule dual inhibitor of c-Met and anaplastic lymphoma kinase (ALK) that was approved for therapeutic use in advanced non-small cell lung cancers positive for either c-ros oncogene 1 (ROS-1) or ALK. Crizotinib inhibits c-Met and ALK by directly binding the activation loop and stabilizing the autoinhibitory conformation of each kinase. Though crizotinib is a potent inhibitor of both kinases, it exhibits greater specificity against c-Met [177].

Preclinical studies using crizotinib alone show potent reduction of proliferation for HNSCC cell lines (cell IC50 4.1–4.7 μM) and a significant inhibition of both wound-closure and invasion in vitro. Crizotinib induced apoptosis and greatly decreased the tumor burden in a patient xenograft in vivo model [120]. In combination with the EGFR inhibitor gefitinib, crizotinib showed greater inhibition of cell survival in vitro as well as impaired wound closure and invasion. Similar results were obtained in vivo with enhanced reduction of patient xenograft tumor volume and the number of cells positive for proliferation markers [170]. Conversely, when crizotinib was combined with radiation therapy, inhibition of c-MET appeared to reduce radiosensitivity in vitro as measured by colony forming assay and in vivo using a mouse model [178]. To date, no clinical trials have been performed using crizotinib to treat HNSCC.

### 9.2. Capmatinib (INC-280)

To date, capmatinib is the only class Ib small molecule inhibitor of c-Met to be used in clinical trials for HNSCC. This class of inhibitors, like class Ia, interacts with the autoinhibitory loop of c-Met, yet they also take advantage of the smaller ATP-binding site of c-Met to generate greater specificity against this kinase [120]. Unfortunately, little in vitro data is available for the effects of capmatinib on head and neck cell lines; however, it is known to be a potent inhibitor of c-Met in a kinase assay (enzymatic IC50 0.13 nM) and proliferation of other tumor lines (cell IC50 1.2–12.4 nM). In tumor-bearing mice, capmatinib was able to inhibit tumor growth and even cause complete regression of some tumor lines without any noticeable toxicity [179]. An ongoing Phase I trial is aimed at determining safety and tolerability of capmatinib in advanced refractory c-Met-dependent solid tumors (NCT01324479). Another Phase Ib/II trial exploring the safety and efficacy of capmatinib treatment with cetuximab, an anti-EGFR antibody, in HNSCC and metastatic colorectal cancers positive for c-Met has recently been suspended for unknown reasons (NCT02205398).

### 9.3. Golvatinib (E7050)

Golvatinib is one of two class II c-Met inhibitors to be used in clinical trials. These inhibitors are large, hydrophobic molecules that occupy the back pocket of c-Met and are usually less specific . As such, golvatinib is a dual inhibitor against c-Met and vascular endothelial growth factor (VEGFR)-2 (enzymatic IC50 14 nM and 16 nM, respectively). In preclinical studies, it inhibited proliferation of a broad range of tumor cell lines (cell IC50 6.2 nM to 4.3 μM) and inhibited HUVEC growth in response to HGF and VEGF as an in vitro model of angiogenesis. Golvatinib also greatly reduced tumor burden in mouse xenograft models and blood vessel density in remaining tumors [180].

Golvatinib has been tested in two clinical trials for HNSCC to date. The first study is a Phase I/II trial targeting EGFR with cetuximab in the presence or absence of golvatinib and is currently recruiting (NCT01332266). The second clinical trial is meant to determine the efficacy of golvatinib treatment in conjunction with cepecitabine and cisplatin (NCT01355302). Unfortunately, this trial did not pass Phase I with five of the seven enrolled patients experiencing serious adverse effects, including supraventricular tachycardia, convulsion, and pulmonary embolism.

### 9.4. Foretinib

Foretinib is a class II inhibitor targeting c-Met, VEGFR2, Recepteur d’Origine Nantais (RON), kinase insert domain receptor (KDR), and Fms related tyrosine kinase 1 (Flt-1) with potent inhibition of c-Met kinase activity (enzymatic IC50 1.16 nM) [181]. In HNSCC cell lines, foretinib alone strongly inhibited cell growth (cell IC50 0.61–0.79 μM) and exhibited synergistic inhibition of cell proliferation when combined with the EGFR inhibitor erlotinib [169]. Foretinib has also recently been demonstrated to contribute to radiosensitization in models of esophageal squamous cell carcinoma. Following irradiation, foretinib-treated cells had diminished repair of DNA double-stranded breaks and underwent more apoptosis. This effect was confirmed in vivo as seen by reduced tumor burden in mouse xenografts [182]. It is unclear why inhibition of c-Met by foretinib increased radiosensitivity, whereas inhibition with crizotinib appeared to have the opposite effect, though this phenomenon is likely due to other kinases targeted by these two molecules.

A clinical trial examining the safety and efficacy of foretinib alone in treating recurrent, metastatic HNSCC was terminated after Phase I after none of the enrolled patients presented partial or complete response (NCT00725764). Though this trial did not progress, enrolled patients had a 50% disease stabilization rate, and 6 of the 14 patients showed shrinking tumor size [183]. No clinical trials to date have used foretinib in combination with other agents to treat HNSCC.

### 9.5. Ficlatuzimab

Ficlatuzimab is currently the only biological therapy undergoing trial that targets the c-Met/HGF axis in HNSCC. It is a humanized monoclonal antibody that sequesters HGF, thereby preventing the stimulation of c-Met by HGF. Given its mechanism of action, however, it is important to recognize that ficlatuzumab is unable to inhibit HGF-independent c-Met activation. Ficlatuzumab has been demonstrated to interfere with stromal contributions to proliferation, migration, and invasion in a preclinical in vitro model of HNSCC, as well as epithelial to mesenchymal transition (EMT) [184]. Ficlatuzumab is currently being tested in combination with cetuximab in a Phase Ib clinical trial of recurrent and metastatic HNSCC (NCT02277197) (Table 1).

## 10. Conclusions

Over three decades of research have been spent investigating the HGF/c-Met pathway and its clinical relevance. In HNSCC, c-Met receptor overexpression is commonly associated with poorer outcomes in patients. This is attributable to increased nodal metastases, cellular proliferation, and cell survival. Notably, c-Met contributes to treatment resistance by bypassing traditionally clinically inhibited signals such as EGF. However, Met inhibitors have yet to show marked improvement for patient outcomes. One of the hurdles to overcome is shifting promising multi-signal blockades from preclinical to clinical trials. Further, there is a need to develop c-Met-specific agents and use these in patients with appropriate biomarkers. Understanding the integration between HGF/c-Met axis and other oncogenic signaling pathways may lead to stronger treatment modalities in the near future (Table 2).

## Figures and Tables

**Table 1:** Inhibitors of the HGF/c-Met axis under investigation in HNSCC.

Compound	Class	IC50/K*_i_*		Co-Treatments	Stage of Development in HNSCC
		c-Met	Cell		
Crizotinib	Ia	2.0 nM (K*_i_*)	4.1–4.7 μM	Gefitinib	Preclinical
				Radiation	Preclinical
Capmatinib	Ib	0.13 nM (IC50)	1.2–12.4 nM	Cetuximab	Phase Ib/II (NCT02205398)
Golvatinib	II	14 nM (IC50)	6.2 nM–4.3 μM	Cetuximab	Phase I/II (NCT01332266)
				Cisplatin and Capecitabine	Phase I/II * (NCT01355302)
Foretinib	II	1.16 nM (IC50)	0.61–0.79 μM	Erlotinib	Preclinical
				Radiation	Preclinical
				N/A	Phase I/II * (NCT00725764)
Ficlatuzumab	Monoclonal Antibody	N/A		Cetuximab	Phase I (NCT02277197)

* Denotes the trial did not progress into phase II. c-Met: mesenchymal-epithelial transition factor; HNSCC: head and neck squamous cell carcinoma.

**Table 2:** Structures of small molecule inhibitors of c-Met.

Crizotinib	Golvatinib
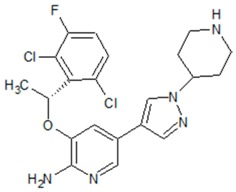	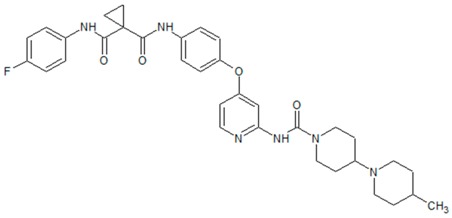
**Capmatinib**	Foretinib
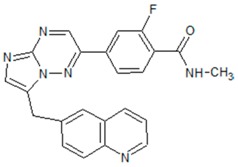	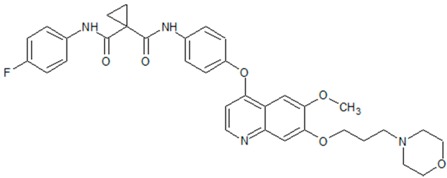

## Abbreviations

EGFR	Epidermal Growth Factor Receptor
c-MET	Met Tyrosine Kinase Receptor, mesenchymal epithelial transition factor
HGF	Hepatocyte Growth Factor
HNSCC	Head and neck squamous cell carcinoma
HPV	Human Papilloma Virus
EGF	Epidermal Growth Factor
mAB	monoclonal antibody
TME	Tumor Microenvironment
SF	Scatter Factor
HSPG	Heparan Sulfate Proteoglycan
